# Les infections nosocomiales en milieu de réanimation: incidence annuelle et aspects cliniques au Service de Réanimation Polyvalente, Kairouan, Tunisie, 2014

**DOI:** 10.11604/pamj.2018.30.143.13824

**Published:** 2018-06-20

**Authors:** Latifa Merzougui, Tarek Barhoumi, Tayeb Guizani, Hafed Barhoumi, Hajer Hannachi, Elyess Turki, Wael Majdoub

**Affiliations:** 1 Service d’Hygiène Hospitalière, CHU Ibn El Jazzar, 3100 Kairouan, Tunisie; 2 Service de Réanimation Polyvalente, 3100 CHU Ibn El Jazzar, Kairouan, Tunisie; 3 Service de Médecine Légale, 3100 CHU Ibn El Jazzar, Kairouan, Tunisie

**Keywords:** Infections nosocomiales, réanimation, incidence, infections associées aux soins, facteurs de risque, Nosocomial infections, resuscitation, incidence, healthcare-associated infections, risk factors

## Abstract

Bien que les réanimations ne comprennent en général qu’une faible proportion des lits hospitaliers; elles constituent la scène d’une forte proportion des infections nosocomiales La gestion du risque infectieux en réanimation constitue aujourd’hui une priorité; l’un des axes stratégiques prioritaires est la mise en place d’un système de surveillance épidémiologique. Nos objectifs étaient de déterminer l’incidence et les Aspects Cliniques afin d’identifier les facteurs de risque. Il s’agit d’une étude descriptive longitudinale d’incidence incluant les patients ayant dépassés 48 heures dans le service de réanimation polyvalente à l’hôpital Ibn El Jazzar de Kairouan sur une période d’une année allant de 01/03/2013 jusqu’au 28/02/2014. L’enquête a porté sur 265 patients dont l’âge moyen était 39±20 ans (18 à 93 ans) et le sexe ratio (H/F) a été de 2,48. Nous avons identifié 125 épisodes d’infections nosocomiales chez 81 malades soit une incidence de 30,6%. La densité d’incidence était de 55 infections pour 1000 jours d’hospitalisation. On a notifié une nette prédominance des pneumopathies avec une incidence de 27,73%, suivies des infections urinaires de 9,73%, des infections liées au cathétérisme veineux central 6,25% et des infections du site opératoire de 2,34%. Le taux de mortalité parmi les enquêtés a été de 28,7% avec une différence significative entre les patients infectés (44,7% des cas) et ceux non infectés (29,07%) (p < 10^-3^). Les microorganismes étaient des BGN dans 80% des cas. La prévention ne peut se concevoir que sous la forme d’une action globale et multidisciplinaire.

## Abstract (en)

Although the number of ICU beds is generally limited, the incidence rates of nosocomial infections is high. Managing infectious risk in ICU is a priority today; one of the strategic aims is the establishment of systems for epidemiological control. The aim of our study is to determine the incidence and the clinical aspects in order to identify the risk factors. We conducted a longitudinal descriptive impact assessment study enrolling patients hospedalized for more than 48 hours in general purpose Intensive Care Unit at the Ibn El JAZZAR Hospital, Kairouan over a period of 1 year, from 01/03/2013 to 28/02/2014. The study focused on 265 patients whose average age was 39±20 years (18-93 years) with a sex ratio(M/F) of 2.48. We identified 125 episodes of nosocomial infections in 81 patients corresponding to a total incidence of 30.6%. The incidence density rate was 55 infections per 1000 days of hospitalization. There was a clear predominance of pneumopathies, with an incidence of 27.73%, followed by urinary tract infections (9,73%), infections associated with central venous catheter (6.25%) and surgical site infections (2.34%). Mortality rate of patients was 28.7%, with a significant difference between infected patients (44.7% of cases) and non-infected patients (29.07%) (p < 10^-3^). Gram-negative bacteria were found in 80% of cases. Prevention must involve a global and multidisciplinary action.

## Introduction

Les unités de réanimation sont considérées comme un réservoir important de bactéries multi résistantes et un endroit où la survenue des infections associées aux soins (IAS) est très fréquente. Ce risque généré directement par la réalisation des soins est favorisé par le caractère invasif des procédures [1]. Une meilleure maîtrise de ces facteurs exogènes devrait permettre de diminuer ce taux de 30% [2 ,3]. La gestion du risque infectieux en réanimation constitue aujourd’hui une priorité; en effet ce risque est bien supérieur à celui encouru par les patients en hospitalisation conventionnelle (13,1% contre 5,4% en France) [1,4]. En Tunisie la prévalence des IAS était de 6,7% en 2005 [5] Les services de réanimation occupaient la première place avec une prévalence des patients infectés de 28,8%. Les résultats sont variables d'un service à l'autre mais les chiffres restent assez élevés, en 2010 Kallel et al. [6] rapporte une densité d'incidence de 34,7/1000 jour-patient alors qu'en 2015 chouchen et al retrouvent une densité d'incidence de 16,9 /1000 jour-patient [7]. Idéalement, chaque établissement de soins doit mettre en œuvre sa propre politique de lutte et de prévention des IAS. Une telle politique doit en principe être fondée en tenant compte des données épidémio-économiques disponibles à l’échelle de l’établissement. Dans notre hôpital, l’un des axes stratégiques prioritaires de lutte et de prévention des IAS est la mise en place d’un système de surveillance épidémiologique. Notre étude s’inscrit dans le cadre de cette politique d’établissement. Elle a le mérite d'être la première enquête d'incidence en milieu de réanimation et de s'étaler sur une période d'une année. Nos objectifs étaient de déterminer le taux d’incidence et la densité d’incidence des IN en réanimation; de décrire le profil épidémiologique et clinique des patients ayant acquis une Infection nosocomiale afin d’identifier ses facteurs de risque.

## Méthodes

Notre étude a été effectuée à l’hôpital Ibn El Jazzar (525 lits); au sein du service de réanimation polyvalente qui comporte 14 lits répartis sur 2 box de 6 lits et un troisième boxe d’isolement de 2 lits. En 2012 Le nombre annuel total des admissions était de 382 avec 3173 journées d’hospitalisation. Il s’agit d’une étude descriptive prospective longitudinale d’incidence incluant les patients hospitalisés dans le service de réanimation polyvalente* sur une période d’une année allant du 01/03/2013 jusqu’au 28/02/2014. Tous les patients hospitalisés dans notre service ayant dépassés 48 heures ont été inclus dans la surveillance de manière ininterrompue que le patient soit infecté ou non (date de sortie ≥ date d’entrée + 2 jours). La date de sortie sert de marqueur d’inclusion. La surveillance du patient cesse une fois le patient est sorti du service ou décédé.

Les définitions opérationnelles que nous avons utilisées sont celles du Comité Français Technique des Infections Nosocomiales et des infections Liées aux Soins "CTINILS" [8]. En fait ce comité reprend en grandes parties les définitions du Center of Disease Control and prevention (CDC). Les colonisations des cathéters étaient exclues. La stratégie de surveillance est basée sur l’approche clinique: il s’agit d’un recueil des facteurs de risque liés au patient, à sa prise en charge et aux complications infectieuses. Notre protocole a été inspiré de celui du réseau national Français d’alerte, d’investigation et surveillance des infections nosocomiales en réanimation REA RAISIN-2013 [1]. Le recueil des données est assuré de façon active et journalière par des enquêteurs préalablement formés (deux résidents en réanimation et un médecin hygiéniste). La validation des fiches d'enquête et des diagnostics retenus est faite par le comité de pilotage (présidé par un professeur agrégé en ranimation et un assistant hospitalo-universitaire en médecine communautaire et préventive); en effet des réunions mensuelles ont été tenues au cours des staffs du service de réanimation pour évaluer et suivre la progression de l'enquête.

L’analyse des données a été réalisée avec un logiciel SPSS version 18 et a permis: 1) Une description du profil épidémiologique des patients surveillés; 2) L’étude de l’exposition aux risques essentiellement en termes d’exposition aux dispositifs invasifs (degrés d’exposition ; durée d’exposition; le ratio d’exposition); 3) **Le ratio d’exposition aux dispositifs invasifs (RDEI):** il tient compte à la fois du pourcentage de patients exposés et de la durée de leur exposition puisqu’il se calcule ainsi (ex: pour le sondage urinaire) Somme des journées de sondage urinaire x 100 / Somme des durées de séjour des patients. Il illustre la proportion des journées d’hospitalisations durant lesquelles les patients ont été exposés à un dispositif invasif donné; 4) **Le ratio d’exposition aux dispositifs invasifs spécifique (RDEIS):** s’intéresse uniquement aux patients exposés à chaque dispositif invasif. (Ex: pour le sondage urinaire): somme des journées de sondage urinaire x 100 / Somme des durées de séjour des patients ayant bénéficié d’un sondage urinaire; 5) Une description des infections surveillées (site, germes, évolution); 5) Le calcul des indicateurs d’incidence (incidence des patients infectés; incidence d’infections nosocomiales; densité d’incidence d’infections). On a utilisé le test de Chi deux pour comparer les pourcentages (Test Exact de Fisher pour les faibles effectifs) et le test de Student pour la comparaison des moyennes. Le risque de premiere espèce a été fixé à α = 5% (le seuil de signification est p < 0,05).

## Résultats

### Caractéristiques de la population d’étude

Durant la période d’étude, le nombre total des patients hospitalisés au service de réanimation polyvalente de l’hôpital Ibn El Jazzar était de 536 malades. Le nombre des patients ayant répondu aux critères d’inclusion était de 265 soit 49,44% des hospitalisés. La traumatologie était le premier motif d’hospitalisation (57,3%) suivie par les pathologies médicales (25,7%). La prise en charge post opératoire des chirurgies programmées et urgentes a occupé la troisième place avec 17% des hospitalisations. L’âge moyen de notre population était de 39±20 ans avec des extrêmes allant de 18 ans à 93 ans. La moitié de la population (46%) avait un âge entre 20 et 40 ans. Notre population était caractérisée par une prédominance masculine. Le sexe ratio (masculin/féminin) était 2,48. L’IGS II moyen de notre population était de 19,26 ± 10 avec des extrêmes allant de 0 à 53. Un score IGS II entre 10 et 30 était retrouvé chez 71,3% des patients (189 malades). La durée d’hospitalisation moyenne était de 8±6 jours (2 jours - 56 jours). Le nombre total de journée passée en réanimation était de 2256 jours.

### Exposition aux dispositifs extrinsèques

Deux cent quarante et sept parmi les 265 malades enquêtés (93,2%) ont eu au moins un dispositif médical invasif. Le ratio de dispositif par patient était de 3,01. Soixante six pour cent des malades (n = 164) étaient exposés en même temps à trois dispositifs médicaux et plus. Parmi les malades intubés, seulement sept patients ont nécessité Une ré- intubation et 20 ont été trachéotomisés. Durant la période d’étude ,187 cathéters centraux ont été utilisés et 30 patients ont eu plus d’un cathéter durant leurs hospitalisations. La voie sous Clavière a été utilisée dans 61% des cas (n =114), la voie jugulaire dans 26% et la voie fémorale dans 13 % des cas seulement (n = 24). Les patients de notre étude ont passé 89,62% de leurs hospitalisations exposés à un dispositif invasif. En effet, sur 2256 journées d’hospitalisation, ils ont passé 2022 journées exposés au moins à un dispositif invasif. Le sondage vésical avait le RDEI et le RDEIS les plus élevés (88,20% et 93,42%) (Tableau 1). Tous les facteurs extrinsèques étudiés étaient liés de façon statistiquement significative à l’infection nosocomiale (Tableau 2).

**Tableau 1:** Durée d'exposition aux dispositifs médicaux invasifs

Dispositif (DM)	Nombre de Jours patients d’exposition	Durée d’exposition au DM (j)				RDEI	RDEIS
		moyenne	Ecart-type	min	max		
Sonde vésicale	1990	7	4	1	38	88,20%	93,42%
Cathéter veineux central	1407	7	4	2	28	63,12%	85,79%
Sonde D’intubation	1260	8	7	1	22	55,85%	74,42%
Drainage thoracique	180	4	1	2	7	7,97%	25,35%

**Tableau 2:** Caractéristiques de la population étudiée et facteurs associées aux infections nosocomiales

Facteurs de risque	Total des patients inclus (n=265)	Patients infectés (n=81)	Patients non infectés (n=184)	Risque Relatif [IC_95%_]	P
Age (moyenne)	39,28±20	41,63±19	38,25±20	---------	0,209
Genre (H/F)	189/76	58/23	131/53	---------	0,946
Immunodépression (n-%)	76 (28,76%)	28(34,56%)	48 (26,08%)	---------	0,208
IGS II (moyenne)	19,26± 10	23,84±9,25	17,25±9,89	6,59 [4,04-9,13]	***<10^-3^***
Chirurgie (n-%)	110 (41,50%)	49 (60,49%)	61 (33,15%)	2,08 [1,43-3,02]	***<10^-3^***
Durée de séjour (jours)	8,53±6	15,31±10,62	5,52±3,59	9,78 [8,01-11,55]	***<10^-3^***
Intubation (n-%)	165(62,26%)	77 (95,06%)	88 (47,82%)	11,66 [4,40-30,89]	***<10^-3^***
Trachéotomie (n-%)	20 (7,54%)	17 (20,98%)	3 (1,63%)	3,22 [2,44 - 4,26]	***<10^-3^***
Durée d’intubation (jours)	4,75±4	11,32±9,99	1,86±1	9,45 [7,17-11,15]	***<10^-3^***
CVC (n-%)	150(56,60%)	75 (92,59%)	75 (40,76%)	9,53 [4,32-21,22]	***<10^-3^***
Durée de CVC (jours)	5,35±5	12,65±9,47	2,14±2	10,51 [8,87-12,15]	***<10^-3^***
Sondage vésical (n-%)	239 (90,18%)	80 (98,76%)	159 (86,41%)	8,70 [1,26-59,97]	***<10^-3^***
Durée de SV (jours)	7,53±7	13,93±9,01	4,70±4	9,23 [7,51-10,94]	***<10^-3^***
Drain thoracique (n-%)	56 (21,13%)	28 (34,56%)	28 (15,21%)	1,97 [1,38-2,79]	***<10^-3^***
Cathétérisme artériel	134(50,56%)	66 (81,48%)	68 (36,95%)	4,30 [2,59-7,13]	***<10^-3^***
Ratio de dispositif par patient	3,01±1,62	4,29±1,11	2,32±1,43	1,97 [1,61-2,32]	***<10^-3^***

### Incidence des infections nosocomiales

Quatre vingt patients parmi les 265 patients enquêtés dans notre étude ont présenté au moins une infection nosocomiale soit une incidence globale de **30,6%**. Cent vingt et cinq (125) épisodes infectieux ont été répertoriés (plusieurs patients ont presenté 2, voir 3 infections nosocomiales). La densité d’incidence des IAS calculée pendant la période d’étude a été de **55 IN pour 1000 jours patients**.

**Figure 1:**
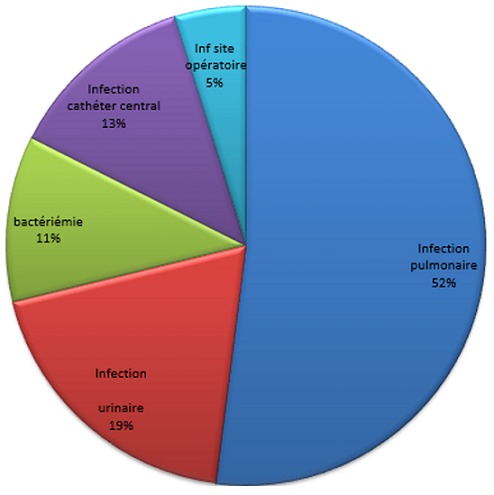
Sites des infections nosocomiales

**Description des infections nosocomiales** Parmi les épisodes infectieux enregistrés dans notre étude, les infections pulmonaires étaient les plus fréquentes avec 71 épisodes soit 57% des IN suivies par les infections urinaires (19%) et les infections liées aux cathéters centraux (13%) (Figure 1). Plus du 1/3 des patients ont contracté 2 infections nosocomiales (35%).

**Pneumopathies nosocomiales:** Soixante neuf épisodes de pneumopathies étaient observés chez 64 patients ventilés mécaniquement et deux épisodes chez deux patients non ventilés. La densité d’incidence était de 54,76 pour 1000 jours de ventilation mécanique. Ces pneumopathies nosocomiales ont été apparues après un délai de 5±3 jours par rapport à l’admission et 4±3 jours par rapport au début de la ventilation mécanique. La confirmation diagnostique a été basée essentiellement sur les résultats des prélèvements distaux protégés dans 70,42 % des cas (n = 50). Les autres épisodes ont été diagnostiqués en se basant sur des critères cliniques, biologiques et radiologiques sans confirmation microbiologique.

**Infections urinaires:** Tous les patients étaient sondés avant l’installation d’une infection urinaire. La densité d’incidence des infections urinaires nosocomiales était de 12 pour 1000 jour de sondage vésical. Le délai moyen d’apparition de l’infection urinaire par rapport au sondage vésical était de 10±2 jours.

**Bactériémies et infections sur cathéters veineux centraux:** La densité d’incidence des infections liées aux cathéters veineux centraux était de 11,23 pour 1000 jour-cathéter. Le délai moyen d’apparition de l’infection par rapport à la pose de cathéter était de 8±4 jours avec des extrêmes allant de 3 à 15 jours. Les bactériémies ont été constatées chez 30 patients .L’origine de la bactériémie était liée à une infection sur cathéter veineux central dans 16 cas, à une pneumopathie et infection urinaire respectivement dans 6 cas, une infection urinaire dans 4 cas aussi et à une infection du site opératoire dans 2 cas. Deux cas de bactériémies primaires étaient identifiés sans foyers associés.

### Documentation microbilogique

Parmi les 125 épisodes infectieux détectés lors de cette enquête, 110 ont pu être documentés (88%). Les microorganismes les plus souvent isolés étaient des bacilles gram négatifs dans 80% des cas. Le *P. Aeruginosa*, la *K. Pneumoniae* et l’*E. Coli* étaient retrouvés dans 66% des cas.

### Evolution

Parmi les 125 épisodes d’infection nosocomiale, 60 ont émaillés de complications: les états de choc de septiques (n = 36); le SDRA (n = 10) et insuffisance rénale aigu (n = 10) et 4 cas de CIVD. Trente huit patients ayant développés au moins une infection nosocomiale ont été décédés (44,7% des cas); cette mortalité est nettement supérieure à la mortalité globale du service durant la même période d’étude (28,7%) et également supérieur à la mortalité chez les non infectés (29,07%) (p < 0,001).

## Discussion

L’infection nosocomiale est le premier événement indésirable en fréquence dans le service de réanimation .Il représente 20 à 30% des complications graves évitables [9]. La surveillance épidémiologique des IAS en réanimation constitue le premier pas dans la lutte contre ce fléau. Cette surveillance permet d’une part d’orienter et de mieux cibler les programmes de prévention et d’autre part de rendre plus aisé l’évaluation des actions de lutte. Notre étude rentre dans le cadre d’une politique de surveillance des IAS en réanimation. C’est un starter pour une maîtrise plus rigoureuse des facteurs de risque, d’écologie bactériennes du service permettant ainsi d’optimiser les différents stratégies de lutte contre ce fléau. Bien que les réanimations ne comprennent en général qu’une faible proportion des lits hospitaliers (< 5%) elles constituent la scène d’une forte proportion des infections nosocomiales à l’hôpital. On estime, en effet, que 20 à 25% de l’ensemble des infections nosocomiales sont acquises dans le secteur de Réanimation dans les différentes études [10]. S’il est acquis que l’incidence des IAS dans une unité de réanimation se situent parmi les plus élevés dans un hôpital, l’importance de ses variations d’une unité à l’autre est une autre évidence. Dans notre enquête, La durée minimale de séjour en réanimation avant inclusion du patient est de 48 h; Le nombre des patients ayant répondu aux critères d’inclusion était de 265 soit 49,44% des hospitalisés; ça pourrait être expliqué par le caractère polyvalent de notre réanimation, en effet vu que c'est la seule unité de réanimation de notre établissement beaucoup des patients sont hospitalisés pour une surveillance post interventionnelle et post traumatique pour des délai ne dépassant pas 48h.

L’incidence des infections nosocomiales était de 30,6%. Nos chiffres sont parmi les plus élevés dans la littérature .En Tunisie, CHAOUCH et al ont montré un taux d’incidence de 29,3%. Au Maroc, Quassimi et al. ont retrouvé un taux d’incidence de 38,4% [11]. Pour les pays occidentaux, les taux sont plus bas. Aux ETATS UNIS, the national nosocomiale surveillance system (NNIS) rapporte une incidence des infections de 9,2%, dans 196 USI [12]. En France, le réseau REA RASIN a conclu à une incidence de 14,1% en 2004 et 15% en 2012 [1,13]. Il est admis que les taux d’infection (exprimé en pourcentage) seuls ont peu d’intérêt. Les travaux du Center for Deseases Control (CDC) ont mis les bases des méthodes de surveillance en montrant l’importance d’exprimer les taux en densité d’incidence et ceci en rapportant les infections à la durée d’exposition (sonde urinaire, cathéter veineux central ou ventilation mécanique…) [14].

Dans notre enquête, la densité d’incidence des IN était de **55 pour 1000 jours patients**. Les résultats sont variables d'un service à l'autre mais restent assez élevé, en 2010 Kallel et al. (6) rapporte une densité d'incidence de 34,7/1000 jour-patient alors qu'en 2015 Chouchen et al. retrouvent une densité d'incidence de 16,9 /1000 jour-patient [7]. Pour les densités relatives à l’utilisation des procédures invasives, nos chiffres dépassent ceux de la littérature. Ainsi, nous comptons 50 pneumopathies pour 1000 jours de ventilation mécanique; 12 infections urinaires pour 1000 jours de sondage vésicale et 11,23 infections liées aux cathéters pour 1000 jours de cathétérisme veineux central. Les résultats observés doivent être interprétés en fonction de la durée moyenne de séjour et la durée moyenne d'exposition aux dispositifs invasifs. Malgré que ces deux indicateurs dans notre étude ne dépassent pas ceux retrouvés dans la littérature [1, 11, 15-18] Nos données d'incidence paraissent les plus élevés (**Tableau 3** et **Tableau 4**).

**Tableau 3:** incidence des infections nosocomiales en Réanimation dans différents études

Etude	Réf	Région-Pays	Année	Période d’étude	Nombre d’unités de réanimation	Nombre de patients	Incidence	Densité d'incidence	Durée moyenne de séjour
Abich	17	Monastir- Tunisie	2003	1 an	1	244	19,8	25,00	8,00
Moalla	18	Sfax- Tunisie	2005	3 mois	1	261	16,9	34,70	8,00
Chaouch	16	Sousse- Tunisie	2010	6 mois	1	215	29,33	20,70	14,00
NNIS	14	USA	1991		31	-	9,20		
Quassimi	11	Maroc	2008		1	147	38,40	35,60	19,00
Réa raisin	1	France	2012		196	29554	13,00	24,16	11,00
Notre étude	--	Kairouan-Tunisie	2014	1 an	1	265	30,6	55,00	8,00

**Tableau 4:** Densité d'incidence des IN selon le site et la durée d'exposition

Auteurs	Réf	Densité d’incidence des PAVM (p/j)	Durée moyenne de VM (jours)	Densité d’incidence d’Inf Ur(p/j)	Durée moyenne de SV (jours)	Densité d’incidence des inf sur CTVC (p/j)	Durée moyenne de CVC (jours)
Abich	17	44,00	-	7,00	-	15,00	-
Moalla	18	22,40	7,00	8,00	8,00	1,00	12,00
Quassimi	11	47,00	14,54	21,00	16,82	16,50	11,28
Chaouch	16	16,60	4,90	11,00	10,7	2,00	7,00
Réa Raisin	1	14,66	9,00	3,85	12,00	0,79	13,00
Notre étude	-	50,00	8,00	12,00	7,00	11,23	8,00

Nos résultats quoi qu'alarmants pourraient refléter la rigueur de la collecte des données (suivi actif et continu), La comparaison doit tenir compte certainement des variations méthodologiques (définition opérationnelle et qualité de collecte des données); mais doit prendre en considération le niveau du maîtrise du risque infectieux dans chaque service. Toutefois cette situation jugée préoccupante pourrait être expliquée par l'absence d'un programme structuré de lutte contre les infections nosocomiales à l'échelle de l'établissement, malgré les efforts des cliniciens réanimateurs qui représentent la catégorie des professionnelles de santé la plus motivée et sensibilisée pour la prévention des IN. En effet notre étude représente le fruit de collaboration entre le service de réanimation et le service d'hygiène hospitalière (ce service a été créé en 2010, il a œuvré à la mise en place d'un programme de lutte contre les IN ayant comme axe stratégique principal: la surveillance épidémiologique se basant sur des enquêtes de prévalence répétitives et des enquêtes d'incidence dans les services à haut risques, ce présent travail est le premier à l'échelle de notre hôpital). Les proportions des différents types d’infections relatives dans notre étude semblent cadrer relativement bien avec celles de la littérature : les pneumopathies viennent en tête, suivies par les infections urinaires et les bactériémies.

Environ 20% des patients nécessitant une ventilation mécanique vont développer une infection pulmonaire [15]. Toutefois, cette incidence varie de 10 à plus de 70% des patients selon les critères diagnostiques utilisés et la population étudiée. Selon le rapport REA RAISIN de 2012, l’incidence des PN globale est de 8,9% et chez les intubés de 11,71%. Entre 2004 et 2010, les PN ont reconnu une baisse de 9,8% [1]. Chaouch et al ont trouvé un taux d’incidence globale des PN de 14% et un taux d’incidence parmi les patients intubés de 28,3% [6]. Pour notre étude, le taux d’incidence global était de 24,52% et 40,64% chez les intubés. L'incidence moyenne Infections Urinaires dans les services de réanimation est élevée de l'ordre de 3,5% à 6,5%. Ces taux, relativement stables depuis le début des années 80, sont 2 à 3 fois plus importants que ceux qui sont rencontrés dans les services de chirurgie et 5 fois plus que ceux des services de médecine [19,20] En France, selon les enquêtes Réa Raisin, l’incidence des IUN était en 2010 et 2012 respectivement de 3,4% et 3,3%. Dans notre étude, l’incidence des IUN était de 7,54%. L'incidence de l’infection liée aux cathéters "ILC" varie selon le type de matériel utilisé, les groupes de patients, le lieu d'hospitalisation, les traitements administrés et le critère diagnostique choisi. Dans les unités de réanimation polyvalente, leur incidence moyenne oscille entre 2 et 10 infections pour 1 000 journées-cathéter, et représente 10% à 25% de l'ensemble des infections nosocomiales, touchant près de 10% des patients hospitalisés en réanimation [21]. En France, le réseau REA RAISIN a notifié en 2012, une incidence de 3,7% et de 0,6% respectivement pour les Bactériémies Nosocomiales et les infections liées au cathéter (ILC). Depuis 2007, l'incidence des BLC a baissé de - 31,3% et celle des ILC de 42,8% alors que la proportion de cultures de CVC positives est restée assez stable [1].

Nos résultats ont montré une incidence des BN de 11,4% alors que l’incidence des ILC était de 6,03%. La densité d’incidence de bactériémies primaires se situe entre 5 et 7/1000 jours-cathéter (11,23/1000 jours dans notre étude). L'incidence des bactériémies liées au CVC est comprise entre 2,8 et 3,2/100 CVC [21]; dans notre étude elle est de 8,6/100 CVC. Nos résultats pourraient être en rapport avec le taux faible d'observance de l'hygiène des mains.

Sur le plan microbiologique, les données de la littérature montrent que 50 à 90% des infections nosocomiales ont une confirmation bactériologique. Dans notre enquête, l’examen microbiologique a été réalisé dans 71% des cas. Les flores responsables d’IN sont en évolution permanente, les incidences respectives des différents groupes bactériens et espèces subissent d’importantes variations, mais les BGN restent prédominantes et représentent 40 à 80% des germes isolés tous sites confondus [22] malgré la recrudescence des CGP et des champignons [23-25]. La flore microbiologique retrouvée dans notre étude est dominée par les Bacilles à Gram négatif: *K. Pneumoniae* (23,6%), *P. Aeruginosa* (20,83%) et *E. coli* (18,05%). Les Cocci Gram positives et surtout le Staphylocoque Aureus occupait seulement 7% de des germes détectés.

La responsabilité directe de l’infection associée aux soins dans la survenue de décès est difficile à établir, en particulier chez des malades polypathologiques, immunodéprimés ou avec des défaillances viscérales multiples mais il est admis que les IN sont à l'origine d’un accroissement de la mortalité et de la morbidité [26]. En Tunisie, rares sont les études qui se sont intéressées à la mortalité imputable aux infections associées aux soins. Une étude menée en l’an 2000 à l’hôpital Farhat Hached de Sousse rapporte une létalité pour les patients surinfectés de 11,8% [2]. Les pneumopathies nosocomiales et les bactériémies sont considérées comme les infections les plus souvent associées aux décès; 24 à 76%pour les pneumopathies et 16 à 35% pour les bactériémies [2,27-29].

La mortalité des infections nosocomiales varie de façon significative avec l’agent responsable. Des taux de 70 à 80% sont observés pour les infections à germes résistants tels que le *Pseudomonas, l’Acinetobacter et le Staphylocoque résistant à la Méticilline* [29]. Il faut souligner que l’existence d’une surmortalité ne préjuge en rien l’évitabilité d’une infection. Ce présent travail nous a permis de faire un état de lieu et un aperçu général sur les IN en Réanimation, malgré quelques limitations concernant essentiellement l'aspect microbiologique (non identification du profil de résistance des germes dans la majorité des cas); le gain essentiel de ce travail était la sensibilisation de tous les intervenants, c'est le starter de mise en place des mesures préventives. Nos résultats ont été présentés et discuter au sein du comité de lutte contre les infections nosocomiales "CLIN"; ils étaient suivi par l'élaboration d'un plan d'action de lutte et de prévention basé sur la promotion de l'hygiène des mains, la maîtrise de l'environnement (désinfection des surfaces, stérilisation des dispositifs médicaux) l'élaboration des procédures et la formation des intervenants. Certes les nouvelles recommandations SRLF/SFAR concernant *les bundles* (association des mesures préventives spécifiques selon le site infectieux) ayant un niveau de preuve suffisant seront prise comme référence dans l'élaboration de nos procédures.

## Conclusion

La mise en place d’une surveillance épidémiologique des infections nosocomiales constitue un préalable obligatoire à toute lutte dans ce domaine, dans la mesure où la surveillance permet d’une part d’orienter et de mieux cibler les programmes de prévention et rend d’autre part plus aisée l’évaluation des actions de lutte. La prévention ne peut concevoir que sous la forme d’une action globale et multidisciplinaire. Ultérieurement une évaluation de nos efforts préventifs se fera à travers une autre étude d'incidence pour démontrer l'efficacité de notre stratégie dans la réduction d'un tel fléau en réanimation.

### Etat des connaissances actuelles sur le sujet

- Les services de réanimation sont des services à haut risque d’acquisition des infections nosocomiales;
- La surveillance épidémiologique est un axe primordial dans toute stratégie de prévention;
- La politique de prévention doit en principe être fondée en tenant compte des données épidémio-économiques disponibles à l’échelle de l’établissement.

### Contribution de notre étude à la connaissance

- Notre étude est la première au niveau de notre établissement à fournir des données d’incidence des infections nosocomiales dans notre réanimation;
- Notre étude a permis d’etudier les aspects cliniques et le profil microbiologique des des infections nosocomiales dans notre réanimation;
- Les resultats de notre étude nous ont permis d’une part d’orienter et de mieux cibler les programmes de prévention et rend d’autre part plus aisée l’évaluation des actions de lutte.
